# Role of Inflammation in Cardiac Remodeling After Acute Myocardial Infarction

**DOI:** 10.3389/fphys.2022.927163

**Published:** 2022-06-28

**Authors:** Francisco A. Fonseca, Maria C. Izar

**Affiliations:** Departamento de Medicina, Escola Paulista de Medicina, Universidade Federal de São Paulo, São Paulo, Brazil

**Keywords:** lymphocytes, C-reactive protein, cardiac magnetic resonance imaging, interleukin-6, acute myocardial infarction

## Abstract

Atherosclerosis is defined as an inflammatory disease. Low-grade inflammation is present in all phases of the cardiovascular *continuum*, since the establishment of cardiovascular risk factors and ischemic heart disease until cardiovascular events, such as myocardial infarction, heart failure and death. Not all inflammatory pathways are linked to cardiovascular outcomes, and thus, not all anti-inflammatory approaches decrease cardiovascular events. The most common cause of ventricular remodeling and heart failure is ischemic heart disease. Biomarkers such as high-sensitivity C-reactive protein can identify individuals at risk of major cardiovascular complications, but this biomarker has no causal effect on cardiovascular disease. On the other hand, interleukin 6 appears to be causally associated with cardiovascular disease. CANTOS was the first proof of concept study showing that anti-inflammatory therapy reduces major cardiovascular outcomes. Based on many anti-inflammatory trials, only therapies acting on the NLRP3 inflammasome, or interleukin 1beta, showed benefits on cardiovascular disease. Ventricular remodeling, particularly after myocardial infarction seems also influenced by the intensity of inflammatory responses, suggesting that anti-inflammatory therapies may reduce the residual cardiovascular risk. Inflammasome (NLRP3) activation, subtypes of lymphocytes, interleukin 6, and some inflammatory biomarkers, are associated with larger infarct size and impaired ventricular function after myocardial infarction. Cardiovascular risk factors commonly present in patients with myocardial infarction, and advanced age are associated with higher inflammatory activity.

## Introduction

Inflammation is present since the earliest phase of atherogenesis (Ross, 1999). Exposure to genetic and environmental factors modulate risk factors and accelerate the development of atherosclerosis (Wen et al., 2022). In the more advanced stages of the disease, inflammation has been recognized and associated with complications of atherosclerosis, such as acute myocardial infarction and stroke (Ridker et al., 1997). After myocardial infarction, infarcted mass and ventricular remodeling also appear to be associated with inflammatory markers (Coste et al., 2020; Casarotti et al., 2021). Recurrent ischemic events have also been associated with persistent low-grade inflammation (Ridker et al., 2018). Finally, anti-inflammatory therapies have reduced major cardiovascular outcomes in patients with established atherosclerosis (Ridker et al., 2017; Tardif et al., 2019; Nidorf et al., 2020). The great challenge today is to act on the residual inflammatory risk without impairing immune responses and with an acceptable long-term safety profile.

## Inflammation and Cardiovascular Risk Factors

**FIGURE 1:**
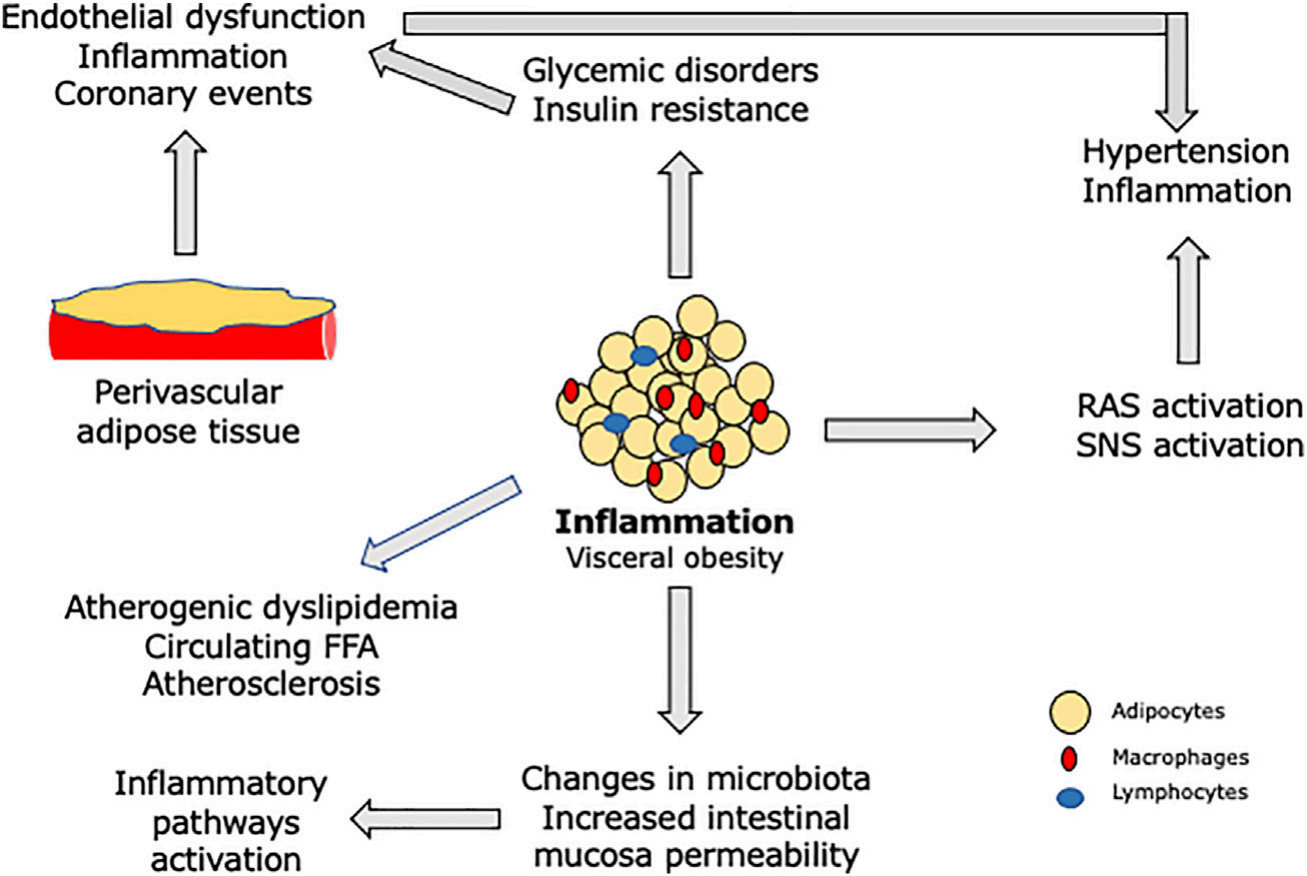
Pivotal role of inflammation in patients with metabolic syndrome. RAS—renin angiotensin system; SNS—sympathetic nervous system; FFA—free fatty acids. Perivascular adipose tissue causes endothelial dysfunction and local inflammation, contributing for atherosclerotic plaque complications. Changes in microbiota composition and higher permeability of the intestinal mucosa to bacterial products such as lipopolysaccharides activate inflammatory pathways. Macrophages and lymphocytes infiltrate visceral adipose tissue and release proinflammatory cytokines, which are related to insulin resistance. These cytokines and FFA change cell signaling related to insulin causing endothelial dysfunction, insulin resistance, and cardiovascular remodeling. The systemic inflammatory status is also related to increased thrombotic risk, leading to ischemic events.

It is estimated that approximately 25% of adult population has metabolic syndrome (Hennekens and Andreotti, 2013). Patients with metabolic syndrome are known to have increased cardiovascular and all-cause mortality (Lakka et al., 2002). These patients have obesity, dyslipidemia, arterial hypertension, and glycemic disorders, associated with peripheral insulin resistance and a systemic inflammatory state (Cercato & Fonseca 2019). Along with visceral fat deposition, an infiltrate of inflammatory cells changes the behavior of adipocytes and release pro-inflammatory cytokines (Rocha et al., 2008). In parallel, increased permeability of the intestinal mucosa allows greater passage of lipopolysaccharides for the circulation (Du et al., 2022). These lipopolysaccharides, after interaction with toll-like receptors, trigger pro-inflammatory responses related to cardiovascular disease, such as interleukin-6 (Saad et al., 2016). All individual components of the metabolic syndrome are interrelated and contribute to a systemic inflammatory state. More recently, metabolic syndrome has been associated with Alzheimer disease (Rojas et al., 2021). Figure 1 shows the main pathways of metabolic syndrome components to inflammation.

## Inflammation and Atherosclerosis

The first step in the formation of atherosclerotic plaques involves the recruitment of inflammatory cells, such as blood monocytes, which differentiate into resident macrophages in the vascular intima, allowing interaction with lipoproteins and formation of the fatty streaks, considered the initial lesions of atherosclerosis (Libby, 2021a). Interestingly, despite the contribution of oxidized low-density lipoprotein (LDL) to the formation of foam cells, antioxidant therapies have failed to prevent the complications of atherosclerosis (Witztum & Steinberg 1991; Shah et al., 2021). However, oxidized LDL also triggers proinflammatory stimuli and it is possible that antioxidant therapy is ineffective in the most advanced phases of the disease, where inflammatory stimuli predominate. In recent years, viral infections such as influenza have been associated with myocardial infarction and other complications of atherosclerosis (Kwok et al., 2015). Influenza vaccination has reduced the risk of main cardiovascular outcomes and has been proposed as part of the therapeutic strategies for patients with coronary heart disease or at high cardiovascular risk (Diaz-Arocutipa et al., 2022; Liprandil et al., 2021).

A fascinating aspect of inflammation occurs shortly after acute myocardial infarction and may explain the high rates of recurrent events in the first weeks and months (Krumholz et al., 2014; Jernberg et al., 2015). Through an experimental model of myocardial infarction, it was observed that the animals developed more pronounced atherosclerotic lesions due to sustained mobilization of inflammatory cells to these lesions (Dutta et al., 2012).

Higher mortality has been observed in older patients with acute myocardial infarction (Helber et al., 2020). Inflamm-aging, a low-grade inflammation in the elderly (Franceschi et al., 2007) appears to contribute to this higher mortality. An imbalance between anti inflamm-aging and inflamm-aging involving higher levels of inflammatory mediators, reduced wound healing, reduced T cell repertoire, impaired innate-adaptive immunity communications, autoimmunity and impaired response to vaccines, among other characteristics were recently reported (Liberale et al., 2022). In addition, clonal hematopoiesis of indeterminate potential (CHIP) due to somatic mutations seems more prevalent in the elderly (1:10 patients over 70 years old). These patients have relatively low risk for development of malignancies, but high risk for atherosclerotic events, establishing a new link between inflammation, aging and cardiovascular disease (Liberale et al., 2022).

## Inflammation and Vulnerable Plaque

For several years, atherosclerosis has been recognized as a progressive, slow-growing disease. However, reports that small obstructive lesions on coronary angiography could progress to myocardial infarction, led to the hypothesis of vulnerable plaques (Ambrose et al., 1988; Arbab-Zadeh & Fuster 2015). In fact, mild obstructive lesions can progress to acute coronary syndromes, but based on angiograms obtained few weeks preceding the coronary event, the average coronary stenosis observed is much more expressive (Ahmadi et al., 2019). Thus, the atherosclerotic plaque growth is now a focus of great interest because preventing the progression of the plaque seems a feasible and crucial step to avoid its complications. Several imaging modalities have been proposed for monitoring the progression of the disease, especially the coronary computed tomographic angiography due to quality of imaging and because it is a non-invasive imaging modality (Dawson et al., 2022). Intensive lipid-lowering and anti-inflammatory strategies have been proposed to prevent plaque progression.

## Inflammation, Myocardial Infarction and Mortality

Inflammatory biomarkers have been related to the prognosis of acute myocardial infarction. Interestingly, higher neutrophil/lymphocyte ratio has been associated with mortality or severity of cardiogenic shock (Jentzer et al., 2022; Tavares et al., 2022). Another available and easily measured inflammatory biomarker is the high-sensitivity C-reactive protein (hsCRP). Plasma levels of hsCRP obtained shortly after myocardial infarction have been related to early and late mortality (Kuch et al., 2008; Alkouri et al., 2022). Based on genetic studies, interleukin 6 (IL-6) appears to have a causal role in coronary heart disease (Swerdlow et al., 2012). Interleukin 6 was also a strong predictor of mortality after acute myocardial infarction (Andrie et al., 2012). In the Canakinumab Anti-inflammatory Thrombosis Outcomes Study (CANTOS), exposure to canakinumab, a monoclonal antibody against interleukin 1 beta, was followed by a significant decrease in the serum levels of hsCRP and IL-6. Compared to patients receiving placebo, those patients under active treatment achieving IL-6 levels below the median had 52% lower cardiovascular mortality and 48% lower all-cause mortality (Ridker et al., 2018).

## Inflammation and Infarcted Mass

Cardiac magnetic resonance imaging (cMRI) has been considered the gold standard method to quantify infarcted mass as it can differentiate transitory to permanent tissue damage, estimation of microcirculation status, and correlates with markers of myocardial injury and inflammatory markers (Mayr et al., 2012). Thus, the effects of markers of inflammation on the infarct size can be adequately evaluated. In the B and T Types of Lymphocytes Evaluation in Acute Myocardial Infarction (BATTLE-AMI) study, the role of circulating interleukins and subtypes of B and T lymphocytes on infarcted mass were analyzed (Fonseca et al., 2017). In the study, compared to the first day of myocardial infarction, there was an increase in the titers of interleukin 4 and interleukin 10, obtained 30 days after myocardial infarction, but the titers of IL-6 did not change overtime (Casarotti et al., 2021). In addition, titers of IL-6 were related to the infarct size (rho = 0.41, *p* < 0.001). In the supernatant of cultured T lymphocytes, there was an increase in the IL-6 titers, explaining why the titers of IL-6 did not change, despite the reduction in the number of circulating T lymphocytes (Casarotti et al., 2021). In the same study, multiple linear regression analysis revealed high-sensitivity troponin T (hsTNT) and IL-6 collected in the first day of myocardial infarction, as independent predictors of the infarcted mass.

## Inflammation and Cardiac Remodeling

**FIGURE 2:**
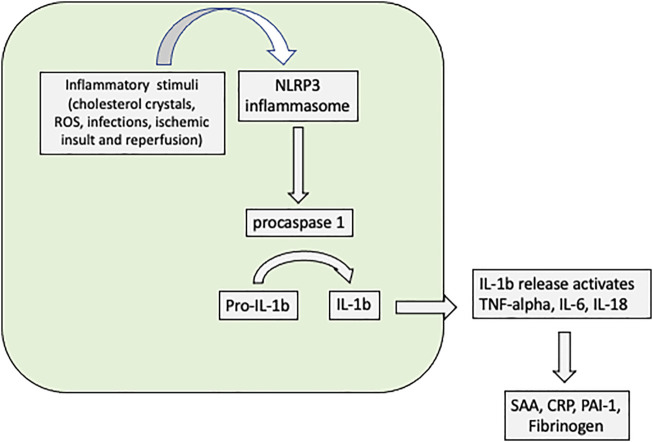
NLRP3 activation triggers the inflammatory pathway of atherosclerosis in monocytes and macrophages resident cells. The ischemic insult due to ischemia and reperfusion after acute myocadial infarction activates the inflammatory pathway mediated by NLRP3. ROS—reactive oxygen species; NLRP3 - nucleotide-binding and oligomerization-like receptor pyrin domain-containing protein 3; IL—interleukin; SAA—serum amyloid A; CRP—C-reactive protein; TNF-alpha—tumour necrosis factor—alpha; PAI-1—plasminogen activator inhibitor type 1. NLRP3 inflammasome is a common inflammatory platform where many stimuli (e.g., cholesterol crystals, ischemic conditions) can activate IL-1beta triggering the inflammatory pathway, including IL-6. The activation of IL-1beta reduces contractility in response to beta adrenergic stimuli and is associated with ventricular remodeling. Further, increase in CRP levels can be used for monitoring of the low-grade inflammation.

Early reperfusion has been recognized as one of the most important strategies to improve outcomes in patients with ST segment elevation myocardial infarction. However, despite timely reperfusion, some patients develop severe myocardial injury and ventricular remodeling with prognosis implications. The nucleotide-binding and oligomerization-like receptor pyrin domain-containing protein 3 (NLRP3) inflammasome seems to have crucial role for atherosclerosis linking cholesterol crystals to inflammation (Duewell et al., 2010). More recently, the inflammatory pathway triggered by NLRP3 seems associated with the myocardial injury after ischemia/reperfusion, affecting ventricular remodeling (Shen et al., 2022). Once activated, NLRP3 inflammasome cleaves procaspase 1 and activates the inflammatory pathway mediated by interleukin 1beta, and subsequently to IL-6 (Figure 2). Thus, activated NLRP3 inflammasome seems related to the myocardial injury after reperfusion, influencing the rates of cell apoptosis and pyroptosis, and the degree of myocardial injury. Experimental pharmacological therapy reducing the NLRP3 activation reported beneficial effect, decreasing myocardial lesion (Mastrocola et al., 2016). In the CANTOS trial, lower rate of hospitalization due to heart failure was observed in patients treated with canakinumab (Everett et al., 2019). In the BATTLE-AMI study (Casarotti et al., 2021), left ventricular ejection fraction obtained by cMRI after 30 days of myocardial infarction was independently related to hsTNT and hsCRP levels collected at the first day of myocardial infarction. Interestingly, B2 lymphocytes (naïve plus memory B cells) collected after 30 days of myocardial infarction were also related to the left ventricular ejection fraction.

## Inflammation and Recurrent Ischemic Events

In the CANTOS trial (Ridker et al., 2017), decrease in inflammatory biomarkers (hsCRP, IL-6) by an anti-inflammatory therapy without changes in cholesterol, glycemia or blood pressure showed a significant decrease in cardiovascular outcomes, constituting proof of concept that residual inflammatory risk in cardiovascular disease can be decreased by an anti-inflammatory strategy. Previously, in the JUPITER trial, the strong reduction observed for cardiovascular events was associated to the lipid-lowering and anti-inflammatory properties of rosuvastatin, but the individualized contribution was not feasible (Ridker et al., 2009). Conversely, in the Cardiovascular Inflammation Reduction Trial (CIRT), the use of low-dose of methotrexate failed to reduce cardiovascular events (Ridker et al., 2019). The trial was stopped after a median of 2.3 years of follow-up without decrease in cardiovascular outcomes. The treatment did not reduce hsCRP, IL-6 or interleukin 1 beta. The study was very important to show that not all inflammatory pathways influence cardiovascular outcomes. Together, the inhibition of NLRP3, interleukin 1 beta, and IL-6 seems the most promising targets for anti-inflammatory therapies. Interleukin 1 induces its own gene expression, and the expression of IL-6 as well. Increased expression of IL-6 further increases acute-phase reactants such as C-reactive protein, fibrinogen, and plasminogen activator inhibitor-1 (PAI-1) (Libby 2021b). Colchicine, another anti-inflammatory drug, showed beneficial effects on cardiovascular outcomes in patients with stable (Nidorf et al., 2020) and acute coronary disease (Tardif et al., 2019). Colchicine, at the molecular level, inhibits NLRP3 inflammasome, in addition to the inhibition of nuclear factor kappa B (Zhang et al., 2022).

## Conclusion

Inflammation is present at all stages of cardiovascular disease, since the development of classic risk factors to the inflamm-aging observed at advanced age.

Persistent low-grade inflammation is related to cardiovascular outcomes and mortality.

Not all anti-inflammatory therapies are effective for cardiovascular disease in reducing cardiovascular events.

The most promising anti-inflammatory strategies involves the lower activation of the inflammatory pathway mediated by IL-6.
